# A Rule-Based Inference Framework to Explore and Explain the Biological Related Mechanisms of Potential Drug-Drug Interactions

**DOI:** 10.1155/2022/9093262

**Published:** 2022-08-17

**Authors:** Adeeb Noor, Abdullah Assiri

**Affiliations:** ^1^Department of Information Technology, Faculty of Computing and Information Technology, King Abdulaziz University, Jeddah 80221, Saudi Arabia; ^2^Department of Clinical Pharmacy, College of Pharmacy, King Khalid University, Abha 62529, Saudi Arabia

## Abstract

As more drugs are developed and the incidence of polypharmacy increases, it is becoming critically important to anticipate potential DDIs before they occur in the clinic, along with those for which effects might go unobserved. However, traditional methods for DDI identification are unable to coalesce interaction mechanisms out of vast lists of potential or known DDIs, much less study them accurately. Computational methods have great promise but have realized only limited clinical utility. This work develops a rule-based inference framework to predict DDI mechanisms and support determination of their clinical relevance. Given a drug pair, our framework interrogates and describes DDI mechanisms based on a knowledge graph that integrates extensive available biomedical resources through semantic web technologies and backward chaining inference, effectively identifying facts within the graph that prove and explain the mechanisms of the drugs' interaction. The framework was evaluated through a case study combining a chemotherapy agent, irinotecan, and a widely used antibiotic, levofloxacin. The mutual interactions identified indicate that our framework can effectively explore and explain the mechanisms of potential DDIs. This approach has the potential to improve drug discovery and design and to support rapid and cost-effective identification of DDIs along with their putative mechanisms, a key step in determining clinical relevance and supporting clinical decision-making.

## 1. Introduction

Drug-drug interactions (DDIs) can result in debilitating illness and sometimes death and so represent serious concerns for pharmaceutical companies, clinicians, and patients worldwide. Indeed, in the US, DDIs were recently found to be responsible for 231,000 emergency room visits [1] and 22.2% of hospital admissions [2]. These numbers illustrate the potential exponential growth of health risks that could occur due to polypharmacy [3], itself a rising concern given the high prevalence of chronic diseases, psychological disorders such as depression, and other such conditions. Interactions such as toxicity or reduced efficacy may occur when two or more agents are coadministered, necessitating dose adjustment or switching to a different therapeutic intervention. While we may prevent additional DDIs by contraindicating drug pairs for which adverse events have been observed in clinic, it remains necessary to develop new methods to improve our understanding of known, unobserved, and potential DDIs [4].

In terms of mechanism, DDIs can be pharmacokinetic (interactions either enhance or reduce effects), pharmacodynamic (interactions occur at or close to the site of action), or both [5]. The mechanisms of most known DDIs were traditionally discovered through single-pathway studies. Examples of traditional discovery approaches include laboratory and animal model studies, as illustrated by the drug interaction guidance document published by the FDA. While this guidance document has enabled researchers and pharmaceutical companies to gain understanding of DDIs, it has some major shortcomings [6]. First, its main limitation is that it focuses mostly on the interactions of cytochrome enzymes (CYPs), ignoring other potential mechanisms. Second, current DDI study requirements are applied during clinical trials; thus, the drugs are often tested in small numbers of patients and in the absence of many possible cofounding factors [7]. As such, the slowness and limited focus of traditional approaches for discovering new and potential interactions means that the problems posed by DDIs will undoubtedly continue.

To address this issue in a cost-effective and high-throughput manner by leveraging machine learning, we proposed a rule-based inference framework to explore and explain the biological related mechanisms of potential DDI mechanisms [8]. First, we designed an extract, transform, load (ETL) method utilizing semantic web technologies to bring together extensive biomedical data, information, and knowledge from diverse resources and integrate it all into a mechanistic knowledge graph. Second, we developed a backward chaining inference rule-based framework to recognize pharmacological and other related mechanistic effects from that graph, applying a set of rules to effectively identify facts that prove and explain the mechanisms of interaction. Finally, we conducted evaluation and validation of the framework using the antineoplastic chemotherapy agent irinotecan and the quinolone antibiotic levofloxacin, whose pharmacokinetics profiles are well-documented.

## 2. Literature Review

Computational methods for predicting DDIs constitute an area of considerable research interest, leading to the development of diverse methods and published resources. These methods have employed a variety of algorithms and utilized many features such as biological effect interactions [9], protein similarities [10], clinical and genomic factors [11], and drug-target [12] and drug-protein [13], as well as drug information on web [14, 15], text-based data [14], protein interaction networks [16], mechanisms of toxicity [17], and enrichment analysis [18]. Among the diverse computational methods that have been used in DDI research, rule-based systems have shown especially promising results [19]. Fundamentally, these systems simulate a human expert's decision-making ability using rules (i.e., IF-THEN statements). For example, a rule-based method has been utilized to discover DDIs from numerous collections of unstructured texts [20, 21]. Others that have been used include knowledge graphs [4, 10], machine learning [22, 23], and deep learning [24].

Despite the quantity of research conducted in this area, recent efforts have realized only limited clinical utility. More specifically, study findings generally only demonstrate associations that are either pharmacokinetic or pharmacodynamic in nature; however, focusing on one level of DDIs neglects important information about other possible interactions such as multipathway interactions [10]. Furthermore, existing efforts have mostly focused on a relatively limited scope of features and have incorporated only a small number of biomedical resources. Still, there is an enormous quantity of data, information, and knowledge available concerning DDIs and their mechanisms of interaction that could be used for exploration [25, 26]. Leveraging these large-scale resources is key to realizing the cost-effective, high-throughput prediction of DDIs that are new to clinical practice or have occult rather than overt effects, as well as diagnosing those that occur in remote treatment situations.

## 3. Methods

### 3.1. Phase 1: The Extract, Transform, Load (ETL) Methodology

In the biomedical domain, data and knowledge are often provided in an assortment of formats, in addition to being provided by a multitude of disparate, disconnected resources; this fractured availability hinders discovery and processing. In the context of DDIs, there is great variability in the reporting of interactions and associated mechanisms by the many commercial and free license resources [27, 28]. Synthesizing these resources to generate a comprehensive utility through which interactions can be discovered both handily and accurately is an ongoing challenge. To overcome these obstacles, we built an ETL method to generate a mechanistic knowledge graph for DDIs. Our ETL method represents an important development towards filling in the knowledge gaps that exist between multiple biomedical resources and ensuring the greater success of knowledge discovery.

The ETL method specifically utilized semantic web technologies for the extraction, integration, and representation of knowledge and data. We also customized it by adding a validation layer that uses reasoning capabilities to test for consistency among classes, instances, and their relationships [29], which is necessary before loading to ensure accurate reasoning. In this phase, the ETL prepares the knowledge graph that will be used later for the inferential task. This phase consists of four main steps: extracting data and knowledge from multiple resources, integrating them into a single graph, validating, and finally loading the information into a knowledge graph in a data store.

#### 3.1.1. Extract: Collecting Information on Drug Mechanisms of Interaction

The first step was to examine data and knowledge resources and extract their relevance to our study, with a focus on information concerning DDIs and their mechanisms of interaction. We mainly examined data and knowledge that contain pharmacological, biomolecular, physiological, and genetic information. Those four groups represented the core classes of the ETL and contained subclasses that categorize the respective instances/entities, i.e., genes and drugs. For pharmacological information, we downloaded DrugBank [30] from https://go.drugbank.com/releases/latest in March 2022. For biomolecular information, we obtained the National Cancer Institute Thesaurus (NCI) [31] and Gene Ontology terms (GO) [32] from the Unified Medical Language System (UMLS) [33]. The National Drug File–Reference Terminology (NDF-RT) [34] for physiological information was also sourced from the UMLS, while the Pharmacogenomics Knowledge Base (PharmGKB) [35], representing genetic information, was downloaded from https://www.pharmgkb.org/downloads in March 2022.

#### 3.1.2. Transform: Building the Mechanistic Knowledge Graph

The transformation phase comprised three major steps:

Step 1: design a mapping strategy for integrating the relevant resources extracted into a single knowledge graph.

Step 2: insert and group relationships between instances and classes in the knowledge graph.

Step 3: choose the appropriate tools to describe and represent entities and relationships among DDIs and their mechanisms of interaction.

For the mapping strategy and the insertion of relationships, we used the UMLS as the backbone for our knowledge graph, as its purpose is to provide an integrated system in the biomedical domain. For semantic tools, we used Jena [36, 37] for building and storing the mechanistic knowledge graph instances and Protégé [38] for creating classes, relationships, and consistency checks.

The process of transformation relied primarily on concept unique identifiers (CUIs) from the UMLS Metathesaurus dataset, which contains more than 4,441,326 concepts and more than 200 relationships from more than 155 biomedical data and knowledge resources. We have written Javascript code using a Jena semantic tool to generate the instances and add their semantic relationships from the UMLS MySQL database into the mechanistic knowledge graph. Specifically, from the MRCONSO table, we extracted and transformed NCI, GO, and NDF-RT resources and then added semantic relationships from the MRREL table. DrugBank and PharmGKB are not part of the UMLS terminology system, so instances and relationships from them were added to the knowledge graph through a shared identifiers technique; for example, the genetic information (relationships) for levofloxacin in the PharmGKB dataset was added to the knowledge graph using the CUI ID link provided at http://pharmgkb.org/chemical/PA450214/link.

#### 3.1.3. Validate: Using the Semantic Network

Validation is always necessary when integrating multiple resources and grouping semantic relationships between instances. Accordingly, we designed an ontology using the Protégé tool, which supports many semantic web formats such as RDF, RDFS, OWL, and XML schema. Four main classes were created to cover the pharmacological, biomolecular, physiological, and genetic levels. After that, we created subclasses as follows: (1) drugs, *rdfs:subclass* of pharmacological; (2) genes, enzymes, and biological processes, *rdfs:subclasses* of biomolecular; (3) effect and mechanism of action, *rdfs:subclasses* of physiological; and (4) SNPs, *rdfs:subclass* of genetic. To properly insert the created instances of the mechanistic knowledge graph into the classes and subclasses for each instance, we utilized corresponding semantic types from the MRSAT table, which contains more than 133 semantic types that together constitute a categorization terminology encompassing diverse biomedical domains. We asserted the semantic types as properties of each instance in the knowledge graph; for instance, T028 represents the Gene or Genome type in UMLS, so all instances that belong to T028 were added to the genes subclass of biomolecular. After validating the knowledge graph, we next checked for consistency among classes, subclasses, and instances, a necessary step when integrating multiple data resources. We used the Pellet [39] reasoner for this task as it is compatible with Jena and wrote Javascript code to check the consistency of our ontology. The results demonstrated it to be appropriately consistent.

#### 3.1.4. Load: Storing the Mechanistic Knowledge Graph in a Triple Store

**Figure 1:**
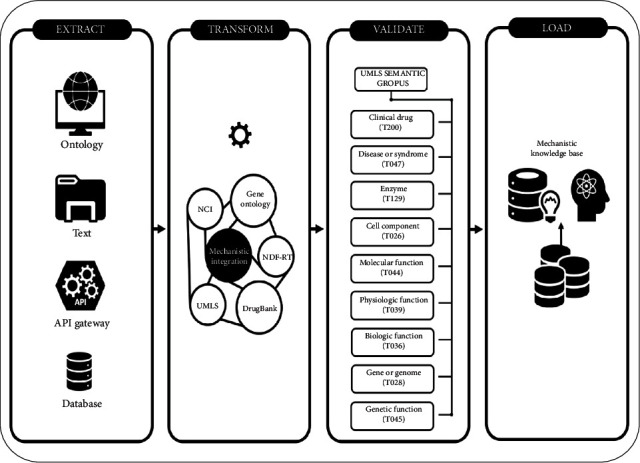
Our methodology for extracting biomedical data, information, knowledge, and features of drugs, transforming them to a knowledge graph, validating the knowledge graph, and then loading them into a comprehensive mechanistic local store.

The last step of the ETL process was to load the knowledge graph into a data store. For this, we used Jena's TDB triple store [37]. The process of creating and uploading the knowledge graph happened once and offline, which allows for fast performance as the information is stored locally with no changes [40].  Figure 1 illustrates the steps of the ETL methodology.

### 3.2. Phase 2: The Inference Algorithm

After creating the mechanistic knowledge graph, we developed the second part of the rule-based framework, the inference algorithm. Rule-based methods have received particular attention in DDI prediction, as they endeavor to mimic the decision-making ability of human experts and have been employed with good results [19], including when extracting information from unstructured text [20, 21]. However, existing systems are relatively limited in terms of both the knowledge resources they draw upon and the types of interactions they consider, and it remains difficult to estimate the actual clinical significance of any given computationally predicted DDI. Leveraging the full breadth of available knowledge [25, 26] and considering multiple and more complex mechanistic dimensions such as multipathway interactions [10] can allow us to not only more comprehensively identify putative DDIs but also assess their relevance to clinical practice. We aimed to do exactly this with our approach.

We used backward chaining as the inference algorithm, which starts with a hypothesis and searches a knowledge graph until that hypothesis is either accepted or rejected [41]. In our study, we hypothesized that there could be a potential interaction between two drugs that have pharmacological effects at the metabolism and transporter levels (i.e., inhibition or induction) and share some biomedical features among four main categories (pharmacological, bimolecular, physiological, and genetic). Given this hypothesis, the framework would then apply rules on a knowledge graph using the backward chaining inference algorithm to find evidence to prove the hypothesis according to a defined set of rules. More specifically, the framework takes two drugs as goals, and the inference algorithm looks for facts in the knowledge graph that return pharmacological effects and shared biomedical features.

## 4. Results

### 4.1. The Rule-Based Framework Requires Multiple Facts and Rules for DDI Exploration and Explanation

**Figure 2:**
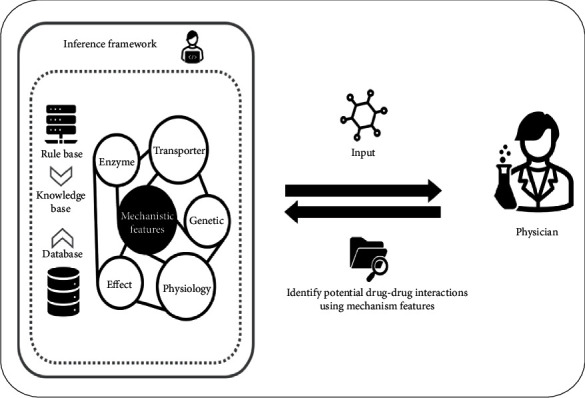
Rule-based framework for exploring and explaining the mechanisms of potential drug-drug interactions.

The framework was developed as summarized in Figure 2. In this framework, we considered a DDI as prospective if it was recognized as having pharmacological effects on the joint basis of either inhibition or induction with respect to metabolism and transport and also shared the maximum number of biomedical features among four core defined categories (pharmacological, bimolecular, physiological, and genetic). We evaluated the framework's performance on a pair of commonly co-administered drugs, the chemotherapy agent irinotecan and the antibiotic agent levofloxacin, for which a potential interaction was previously identified [42].

To explore the possibility of a DDI, we proposed a backward chaining algorithm based on a rules framework that links facts (i.e., IF parts) to conclusions (THEN parts). The developed framework is comprised of six rules, which have been validated by a clinician, to explore and explain the mechanisms of potential DDIs, which are as follows:

### 4.2. Integration of Mechanistic DDI Information and Pharmacological, Biomolecular, Physiological, and Genetic Effects Provide the Necessary Evidence for DDIs in the Clinical Setting

**Figure 3:**
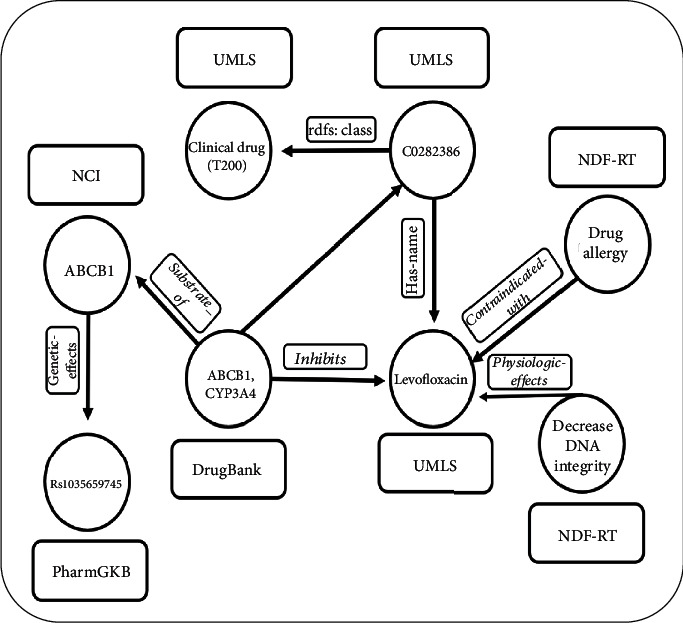
The multiple mechanistic features of levofloxacin, extracted from different biomedical resources.

In our rule-based framework, we combined six types of biomedical data and information (genes, proteins, biological processes, molecular function, physiology, and SNPs) and achieved a high-quality knowledge representation from which to determine whether a potential DDI may exist. A SPARQL query [40] over the mechanistic knowledge graph was generated in which all features of interactor 1, levofloxacin, were retrieved from multiple resources and incorporated in the final framework as demonstrated in Figure 3.

### 4.3. The Framework Proves Potential DDI through Mechanisms of Interaction

To demonstrate the reliability and effectiveness of the implemented rules, we had the framework consider two major layers of backward chaining inference in a sequence that would propose mechanisms for the possible DDI between concurrently-administered levofloxacin and irinotecan. The first layer addresses when both interactors intervene pharmacologically at the metabolism level (i.e., inhibit or induce enzymes and transporters), and the second layer when both interactors share a maximum number of features within the classes (pharmacological, bimolecular, physiological, and genetic). Through conducting backward chaining inference on the knowledge graph, our framework identified the most relevant pathways connecting the two drugs and yielded a proposed mechanism of their DDI, illustrated in Table 1. Specifically, the framework identified levofloxacin as an inhibitor for both an ABC transporter protein, ABCB1, and the CYP3A4 enzyme, while irinotecan was likewise identified as a substrate for both. In humans, CYP3A4 is the most widely expressed of CYP proteins; it constitutes as much as 70% of gastrointestinal CYP activity and mediates 40-45% of all phase 1 metabolic reactions [43–45]. In addition, similarity between the two drugs was detected at three levels, including that both agents are contraindicated when a patient is allergic to either and both decrease DNA integrity, which satisfies the backward chaining rules. Furthermore, the two drugs share substructures; that is, both contain the following: hydroxy compounds, heterocyclic compounds, aromatic compounds, phenols and derivatives, pyridines and derivatives, benzene and derivatives, carboxylic acids and derivatives, acetates, ethers, aliphatic, and aryl amines, phenyl esters, anisoles, (iso)quinolines and derivatives, and hydroxyquinolines.

Therefore, the framework detected CYP3A4 and ABCB1 as primary candidates for the mechanism of this drug pair's interaction. Notably, CYP3A4 and ABCB1 are coexpressed in the liver and intestines, and the liver is where irinotecan is converted to its active metabolite, SN-38, through hydrolysis by carboxylesterases (CESs) [46, 47]. In the liver, SN-38 can be glucoronized, detoxified by enzymes of the UGT1A1 family, and eliminated via release into the intestines; or it can be oxidized through the action of CYP3A proteins. Meanwhile, irinotecan can itself be oxidized by CYP3A proteins, producing either of two inactive metabolites. Ultimately, irinotecan, its elimination, and the abundance of SN-38 are regulated heavily through CYP3A4 and UGT1A1 [46, 47]. In contrast, levofloxacin metabolism in humans is limited; the drug is primarily excreted through the urine without any alteration [48, 49]. Interestingly, the findings from our computational approach suggest a potential effect of levofloxacin on CYP3A4 activity; namely, it could influence the metabolism dynamics of CYP3A4 substrates such as irinotecan. This merits further investigation to improve our understanding of levofloxacin and its DDIs.

Another finding highlighted ATP-binding cassette transporters as a potential candidate mechanism. These transporters comprise a large superfamily of membrane proteins. ABCB1 in particular (along with its analogs) has become known for its importance in the absorption of drugs and drug candidates. Interestingly, numerous reports have found that coadministration of an ABCB1 inhibitor and substrate can greatly increase blood levels of the substrate, resulting in serious side effects [50, 51]. Accordingly, ABCB1 interactors need to be investigated in terms of both their substrate and inhibitor properties—perhaps especially the latter in the context of DDIs. Notably, the literature supports levofloxacin as an ABCB1 substrate [48], while our framework indicated a potential inhibitory effect on ABCB1 transporters; both imply potential interaction of levofloxacin with all ABCB1 substrates, including irinotecan.

Several drugs are known to interact with irinotecan at different levels [42, 52]. Limited data suggest that oral quinolone antibiotics may have their plasma concentrations reduced upon chemotherapy with antineoplastic agents [48, 49]; however, there has not yet been any report of an interaction between irinotecan and levofloxacin. As a broad-spectrum antibiotic, levofloxacin is used extensively in cancer patients for treating infections; in addition, according to the guideline, levofloxacin is also used as a postchemotherapy course prophylactic for patients in which febrile neutropenia is a substantial risk [53]. If levofloxacin and irinotecan interact, extensive use of levofloxacin in patients receiving irinotecan would predispose them to that DDI, which may result in unwanted and potentially hazardous adverse effects.

## 5. Discussion

In this research, we developed a computational framework that utilized a rule-based approach to explore and explain the biological related mechanisms of potential DDIs. This present work focuses strongly on leveraging multiple large-scale biomedical resources to provide support for assessing the mechanisms of interaction at work in DDIs so as to identify potential DDIs. Further, we tested the framework by examining the putative DDI between irinotecan (an antineoplastic chemotherapy agent) and levofloxacin (a quinolone antibiotic); the mechanisms so identified suggest directions for confirming the clinical significance of this predicted DDI.

Upon validation, our framework has the potential to add significant value to current practices surrounding DDIs. First, requirements to identify potential DDIs have been established by food and drug administrations in several countries and are mandatory for new drug approval [6, 54]. Such requirements might increase the cost of drug research and delay approval. Second, the limited knowledge available to clinicians regarding existing and unknown DDIs affects clinical decisions, especially when alternative agents are not available. The mechanism-centered approach employed in this framework allows for consideration of not only a DDI's possible occurrence but also its clinical relevance.

In having developed this framework, it has become clear that the state-of-the-art has mainly focused on a relatively limited scope of features and reasoning and has incorporated a relatively small number of knowledge resources. In existing knowledge of drug mechanisms, biomedical features are among those considered as possible causes; accordingly, if for a pair of drugs known to produce a DDI there is suspicion that a particular set of biomedical features is the cause of that DDI, then it seems reasonable to extend this concern to a hypothetical other pair of drugs sharing the same biomedical feature pattern. Our current framework uses a simplistic form of combined similarity computation that could be greatly expanded on or even replaced in further works. The present implementation considers each biomedical feature pattern to be equally important and therefore implies that the more of these patterns that are satisfied, the more likely there is a need to be concerned about a DDI. It is important to consider how “similar” two pairs need to be to raise an alarm for potential DDI. Under the current framework, the similarity does not need to be very great, and yet the number of false alarms raised seems to be reasonably low. We believe that the basic premise is sound, but enhancement/replacement of the similarity determination with more comprehensive computational methods could yield promising results; we have several thoughts in that direction. Nonetheless, this work fulfills its purpose, which is to provide a new direction as a means of differentiation from prevailing approaches and thereby invite greater attention to be given to the process of evaluation as opposed to the production and expansion of resources for use in evaluation [55].

## 6. Conclusion

This work introduces a rule-based framework that has utility for exploring and explaining possible DDIs, as demonstrated by our case study of the commonly coadministered chemotherapy agent irinotecan and antibiotic levofloxacin. It represents an initial step toward developing an efficient system that can be utilized by researchers and clinicians to reduce requirements for drug approval, particularly concerning DDI studies, and hence accelerate a drug's approval. In addition, such a framework would provide support that aids clinicians in making clinical decisions, especially for new drugs with limited evidence.

## Figures and Tables

**Algorithm 1:**
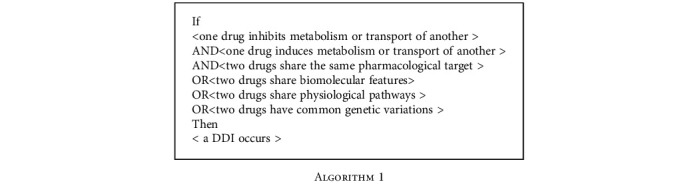


**Table 1:** Summary of the potential mechanism of interaction of coadministered irinotecan and levofloxacin.

	Irinotecan	Levofloxacin
*Pharmacological effects*		
Metabolizing enzymes	CYP3A4 substrate	CYP3A4 inhibitor
Transporters	ABCB1 substrate	ABCB1 inhibitor
*Biomedical features*		
Pharmacological (MoA)	NA	NA
Biomolecular	NA	NA
Physiological	Decreased DNA integrity	
Genetic	NA	NA

## Data Availability

Data is available upon request.
